# Accelerating Adaptation of Forest Trees to Climate Change Using Individual Tree Response Functions

**DOI:** 10.3389/fpls.2021.758221

**Published:** 2021-11-23

**Authors:** Valérie Poupon, Debojyoti Chakraborty, Jan Stejskal, Heino Konrad, Silvio Schueler, Milan Lstibůrek

**Affiliations:** ^1^Faculty of Forestry and Wood Sciences, Czech University of Life Sciences, Prague, Czechia; ^2^Federal Research and Training Centre for Forests, Natural Hazards and Landscape, Department of Forest Growth, Silviculture & Genetics, Vienna, Austria; ^3^Federal Research and Training Centre for Forests, Natural Hazards and Landscape, Department of Forest Biodiversity and Nature Conservation, Vienna, Austria

**Keywords:** assisted migration, genetic diversity, intraspecific variation, provenance trials, European larch

## Abstract

In forest tree breeding, assisted migration has been proposed to accelerate the adaptive response to climate change. Response functions are currently fitted across multiple populations and environments, enabling selections of the most appropriate seed sources for a specific reforestation site. So far, the approach has been limited to capturing adaptive variation among populations, neglecting tree-to-tree variation residing within a population. Here, we combined the response function methodology with the *in-situ* breeding approach, utilizing progeny trials of European larch (*Larix decidua*) across 21 test sites in Austria ranging from Alpine to lowland regions. We quantified intra-population genetic variance and predicted individual genetic performance along a climatic gradient. This approach can be adopted in most breeding and conservation programs, boosting the speed of adaptation under climate change.

## 1. Introduction

Global temperature is likely to increase up to 1.5–2°C by the end of the century (Lindner et al., 2014; Pachauri et al., 2014) along with an increased frequency and intensity of extreme events (Dai, 2011; Trenberth et al., 2014; Seidl et al., 2017; Senf and Seidl, 2021). Due to global warming and the increased atmospheric CO_2_, there is a positive trend in overall forest productivity, mainly when water restriction is not a limiting factor (Boisvenue and Running, 2006). While the above boost in productivity is anticipated in Northern and Western European regions, the Southern counterparts seem more threatened by intensified drought events that may decrease survival and productivity (Lindner et al., 2010). Climate is one of the primary factors influencing local adaptation (Howe et al., 2003; Savolainen et al., 2007). Faced with unfavorable changes in environmental conditions, tree populations can either persist, migrate or go extinct (Aitken et al., 2008). Both persistence and natural migration rates may not sufficiently cope with the predicted rate of climate change (CC) (Davis and Shaw, 2001; Malcolm et al., 2002; McLachlan and Clark, 2004; Richter et al., 2012; Dyderski et al., 2018). Therefore, to reduce the impact of CC on forests, there is an urgent need to understand and secure genetic variation to support future adaptation.

Tree species are known for high levels of genetic variation across vast geographical ranges; genetic differences are observed at different hierarchical levels (among species, populations, to individual trees). Additive genetic variance ($\sigma_{a}^{2}$) is a product of respective allelic frequencies and their direct biochemical effects on individual phenotypes (Falconer and Mackay, 1996). The genetic rate of adaptive response to natural selection (i.e., the directional change in fitness) is directly attributable to the current $\sigma_{a}^{2}$ (Fisher, 1930). Genetic variation in forest trees has been substantially investigated in line with evolutionary forces, human intervention and environmental change, and explored in tree breeding programs for over a half-century, even though selection in these programs has primarily focused on economically important traits (e.g., height, straightness, or disease resistance) (White et al., 2007). CC may imposes a severe constraint to the directional natural (and artificial) selection. Assisted migration could speed up the selection response in adaptive traits (Gougherty et al., 2021; Sáenz-Romero et al., 2021; St-Laurent et al., 2021).

Since the 90s, apart from conventional tree breeding, the correlation of quantitative traits with climatic variables has been studied in several economically important tree species using pre-existing provenance trials (Mátyás, 1994; Schmidtling, 1994; Rehfeldt et al., 1999). Typical provenance trials are composed of specific test sites where several populations (provenances) originating throughout the species range are planted. Response functions have been applied on provenance trial data to investigate the intraspecific genetic adaptation to climatic conditions. Two types of functions have been used: (i) the Transfer Function (Mátyás, 1994; Thomson and Parker, 2008; O'Neill et al., 2014), and (ii) the Response Function (Rehfeldt et al., 1999; Wang et al., 2006; Kapeller et al., 2012). The former is used to analyze the performance of several provenances in a specific environment. The latter tests the performance of a particular provenance across a range of planting conditions across different sites. Thus, transfer functions are based on the genetic drivers, and response functions are based on the environmental drivers of variation in fitness-related traits. Combined with future climatic scenarios, these functions can provide estimates of the impact of CC on forests. They can be used as a decision support tool for seed delineation zones and assisted migration (Leites et al., 2012).

As outlined above, response functions have been proposed to capture adaptive variation at the population (provenance) level, unlike the conventional breeding focusing mainly on intra-population $\sigma_{a}^{2}$. We combine genetic evaluation and the response function methodology to capture intrapopulation adaptive response across environmental gradients. We demonstrate the methodology using progeny trials of European larch (*Larix decidua*) from 21 test sites in Austria ranging from Alpine to lowland regions. We utilized height and wood density measured directly in forest stands on individual mature trees with reconstructed pedigree. Using the response function methodology combined with mixed-model genetic evaluation, we quantified the intra-population $\sigma_{a}^{2}$ matching specific genetically adapted trees to specific climatic variables. This approach can be adopted in most forest tree species, boosting the speed of adaptation under CC while overcoming the practical limitations of traditional breeding and conservation programs.

## 2. Materials and Methods

### 2.1. Plant Material

**Figure 1:**
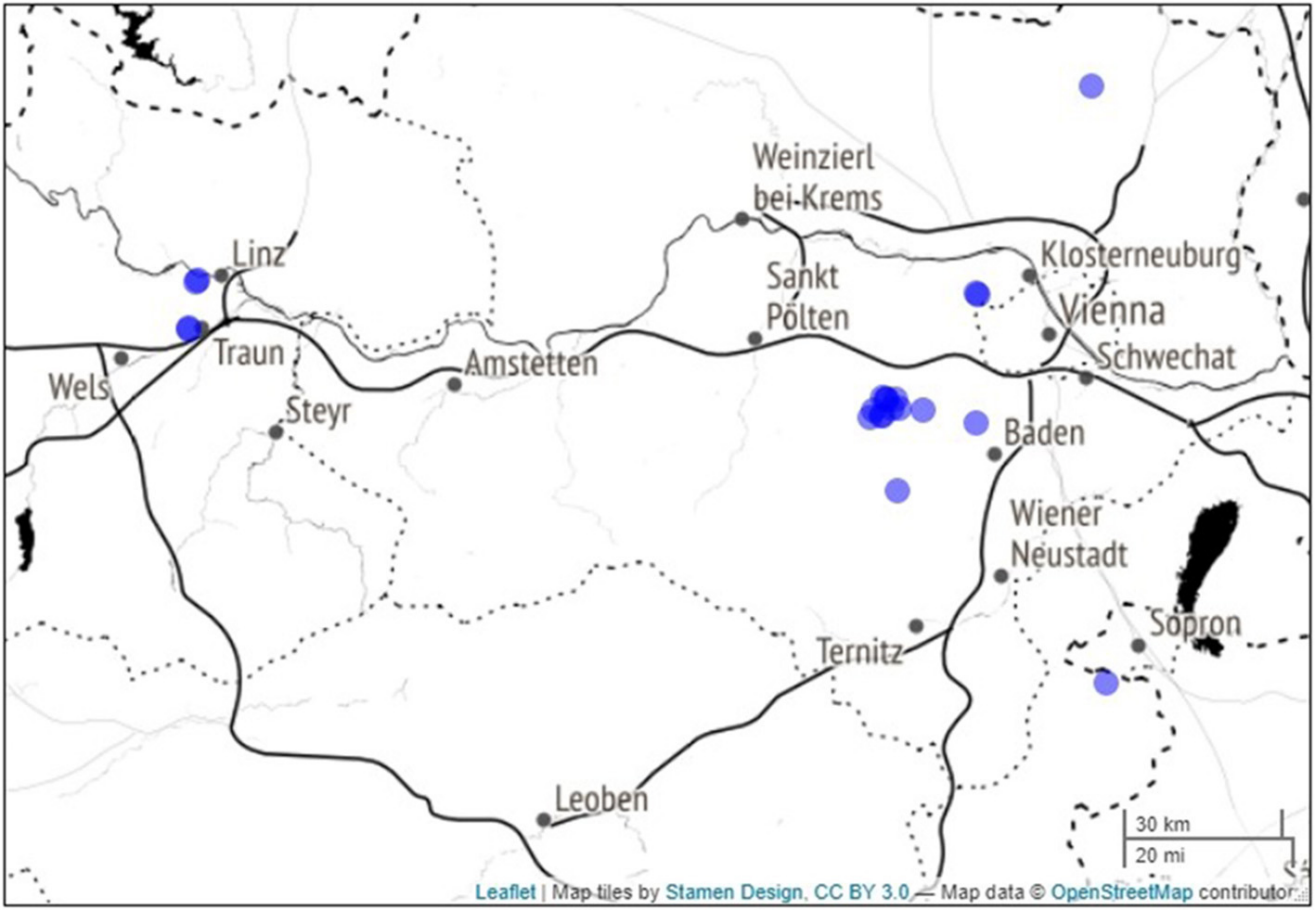
Map of the 21 testing sites (blue dots) spread accross North-Eastern Austria. A darker blue color means that sites are overlaping at this scale.

The European larch data used in this study were previously sampled and genetically analyzed by Lstibůrek et al. (2020). We will briefly outline their methodology related to the current investigation. Originating from a local European Larch provenance, a clonal seed orchard was established in 1954 in the Northern Alpine region by grafting 53 phenotypically superior parental trees. The orchard has served as a major seed source for afforestation activities in the region. Newly established forest stands comprise individual half-siblings, i.e., offspring from random mating (open-pollination) among parental trees in the orchard. One-half of the respective parentage (i.e., paternal gametic contributions) has originated from unknown trees within the orchard. In 2018, 21 of these forest stands (Figure 1, OpenStreetMap contributors, 2021) were selected for phenotyping and genotyping, yielding potential breeding candidates (25 to 37 years old). All sites were situated at altitudes ranging from 280 to 760 m. Over 4,000 individuals were measured for height and wood density, and 1,253 of them, plus the 53 parental trees, had their DNA extracted for microsatellite analysis. A pedigree was then reconstructed using the likelihood-based method implemented in the Cervus software (Marshall et al., 1998). The investigation revealed a marginal 8.4% of parental contributions outside the orchard (i.e., pollen contamination). In total, 491 full-sib families were assembled, representing 35% of the possible 53-parent half-diallel mating scheme. This assembled pedigree (Lstibůrek et al., 2020) constitutes the basis for our subsequent analyses.

### 2.2. Climatic Data

We extracted climatic variables for the test sites from the WORDCLIM dataset (Hijmans et al., 2005). The dataset covers a period from 1950 to 2000 with a 1km spatial resolution of 1 km. It includes monthly temperature means, minima, maxima, monthly precipitation sums, seasonal and annual temperatures, and precipitation variables. Secondly, we used the random forest model (Breiman, 2001) to identify the most important variables. The random forest provides two types of importance measures, the mean decrease in accuracy and the mean decrease in node impurity (Liaw and Wiener, 2002). We selected the most recurring variables over several runs of the model. Afterward, we checked these variables for collinearity, and we plotted preliminary response functions (as explained in section 2.4) to single out the most important variable.

### 2.3. Genetic Evaluation

We conducted all statistical analyses in R (R Core Team, 2020) and Rstudio (RStudio Team, 2020). We utilized the mixed-model genetic evaluation protocol implemented within the ASReml-R (Butler et al., 2017). Individual tree height was divided by the respective stand age to obtain mean annual increment (MAI) values comparable across all sites. MAI phenotypic values were jointly analyzed with wood density in the bivariate animal genetic model following the original protocol by Henderson (1984).


(1) $$\begin{array}{l} \text{y}=\text{X}\text{b}+\text{Z}\text{a}+\text{e} \end{array}$$

where **y** is the vector of bivariate phenotypic observations; **X** is the incidence matrix for the fixed effect **b** (trait and site means); **Z** is the genetic relationship matrix; **a** is the vector of additive genetic (breeding) values, $\text{a}\sim N\left(0,\sigma_{a}^{2}\right)$, and the random residual effects are distributed as $\text{e}\sim N\left(0,\sigma_{e}^{2}\right)$. The covariance matrix for the random additive genetic effects was modeled using the heterogeneous covariance structure as


(2) $$\begin{array}{l} \sigma_{a}^{2}=\left[\begin{array}{cc} \sigma_{a_{1}}^{2} & \sigma_{a_{1}a_{2}}\\ \sigma_{a_{1}a_{2}} & \sigma_{a_{2}}^{2} \end{array}\right]⊗\text{A} \end{array}$$

where **A** is the average numerator relationship matrix, σ_*a*_1_*a*_2__ is the additive genetic covariance between traits 1 and 2, and ⊗ is the Kronecker product operator. The random residual error effect was modeled using an unstructured covariance matrix structure as


(3) $$\begin{array}{l} \sigma_{e}^{2}=\left[\begin{array}{cc} \sigma_{e_{1}}^{2} & \sigma_{e_{1}e_{2}}\\ \sigma_{e_{1}e_{2}} & \sigma_{e_{2}}^{2} \end{array}\right]⊗\text{I} \end{array}$$

where σ_*e*_1_*e*_2__ is the residual covariance between the two traits. Random effects were assumed to be independent.

We utilized the above predictions of the fixed site effects and calculated the respective all-pairwise differences. Next, we trimmed the dataset so that each half-sib family was represented in at least six sites to achieve even representation of families while maximizing their distribution across multiples sites (see Kapeller et al., 2012; Foff et al., 2014; Suvanto et al., 2016 for a similar number of test sites). For each individual, we calculated the predicted phenotypic performance for the MAI (further denoted as PMAI) as a sum of the overall mean, respective site effect, and the individual additive genetic (breeding) value (BV) from the bivariate additive genetic model.

### 2.4. Response Function

We developed individual- and population-level univariate response functions (RF) to describe the within-population genetic variation following major climatic gradients. We tested the linear, quadratic, and Gaussian models as they have been predominantly used in previous studies (Wang et al., 2006; O'Neill et al., 2007, 2014; Leites et al., 2012; Sáenz-Romero et al., 2017). The linear model did not yield a significant fit. Contrastingly, both the quadratic and Gaussian models showed significant fit. The shape of the response function was almost identical, and so was Akaike Information Criterion (AIC). Below, we present the quadratic model that we selected.


(4) $$\begin{array}{l} v_{jk}=\beta_{0}+\beta_{1}c_{j}+\beta_{2}c_{j}^{2} \end{array}$$

where *v* is the estimated mean height at the site *j* for parent *k*; β_0_, β_1_, and β_2_ are regression coefficients; *c* is the climatic variable at planting site *j*.

## 3. Results

Using the bivariate animal genetic model, we obtained significant narrow-sense heritability for height (*h*^2^ = 0.27, SE = 0.07) and wood density (*h*^2^ = 0.30, SE = 0.07), respectively. We observed negligible additive genetic correlation between the respective traits (*r*_*a*_ = 0.09, SE = 0.20). Our data did not show a significant genotype by environment interaction (GxE), as shown earlier by Lstibůrek et al. (2020). Summary of the model fit statistics (full and reduced model) are provided in Supplementary Table S2.

**Figure 2:**
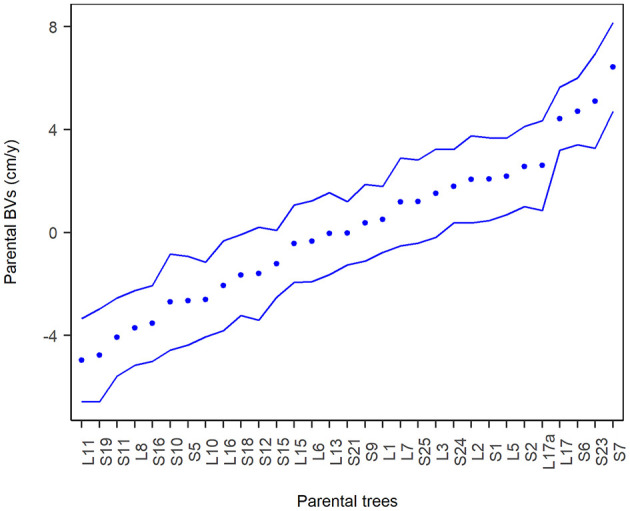
Parental trees are sorted by additive genetic values. The BVs (dots) are expressed in the units of measurement (cm/year). 95% approximate confidence intervals (dashed line) were calculated as two times the standard errors.

Experimental site effects (considered fixed) were found statistically significant (*p* < 0.01). 87% of all pairwise differences between sites were statistically significant (*p* < 0.05). Details are provided in Supplementary Table S1. In Figure 2, BVs for MAI are plotted with their respective confidence intervals showing the extent of additive genetic variation present within a random set of 31 parents. The BVs are ranging from –5 to 6.4 cm (average BV is zero).

**Figure 3:**
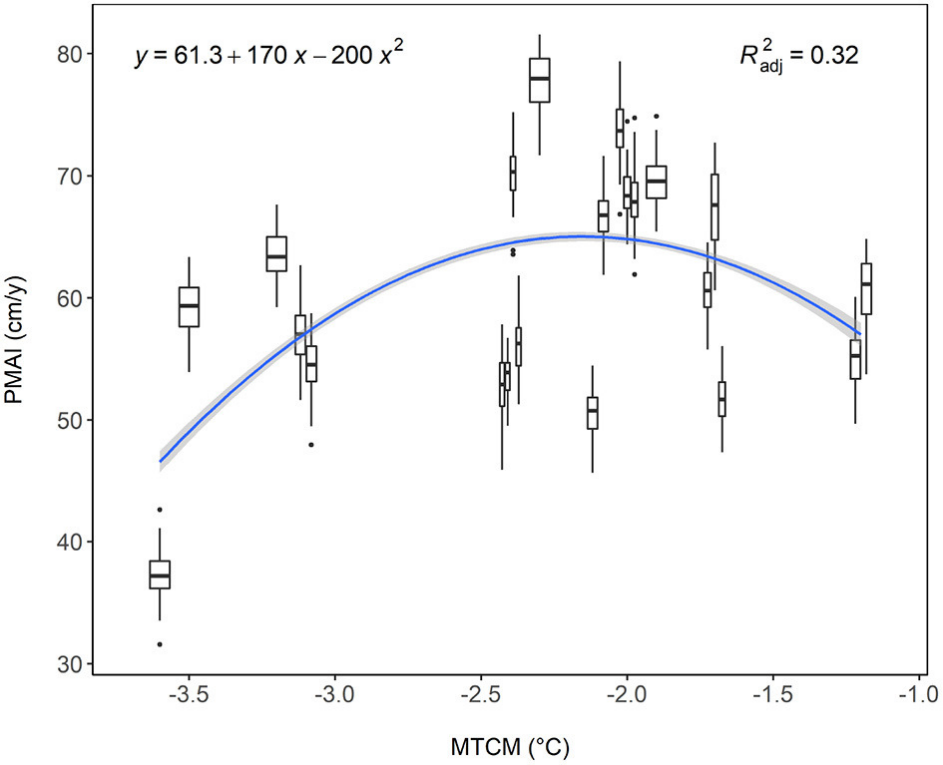
Population RF with $R_{adj}^{2}$ of 0.32. PMAIs are plotted against the sites' MTCM. Boxplot were plotted for each site. The black dots represent the outliers. The gray band represents the curve's 95% confidence interval. Sites are from the left to right: B16, B18, B11, B12/B20, B6/B7/B5/B13, W3, B4/B3, A/B9/N, W4, B2/B1/H2, T1/T2 (sites separated by a “/” have the same MTCM but are plotted next to each other for a clearer plot).

For the response function modeling, the seven variables with the highest importance were selected (Supplementary Table S3): altitude, minimum temperature of January and December, mean temperature of the coldest month (MTMC), mean temperature of December, and maximum temperature of January and December. All of them were highly collinear, with pairwise correlations > 0.85. The preliminary response functions showed similar results for each variable. Finally, we decided to retain only the MTCM, as it explains over 69% of the variability in our data according to the Random Forest model. Following the regression analysis, the adjusted coefficient of determination $R_{adj}^{2}$ in the quadratic model for the RF at the population level was 0.32% with *p* < 0.001 (Figure 3). The PMAI culminates at 65 cm/year for a MTCM of -2.2°C. The 21 boxplots represent the range of families' PMAIs at each testing site. For example, we can see the boxplot of site B16 with both the lowest MTCM and PMAIs values in the lower-left corner.

**Figure 4:**
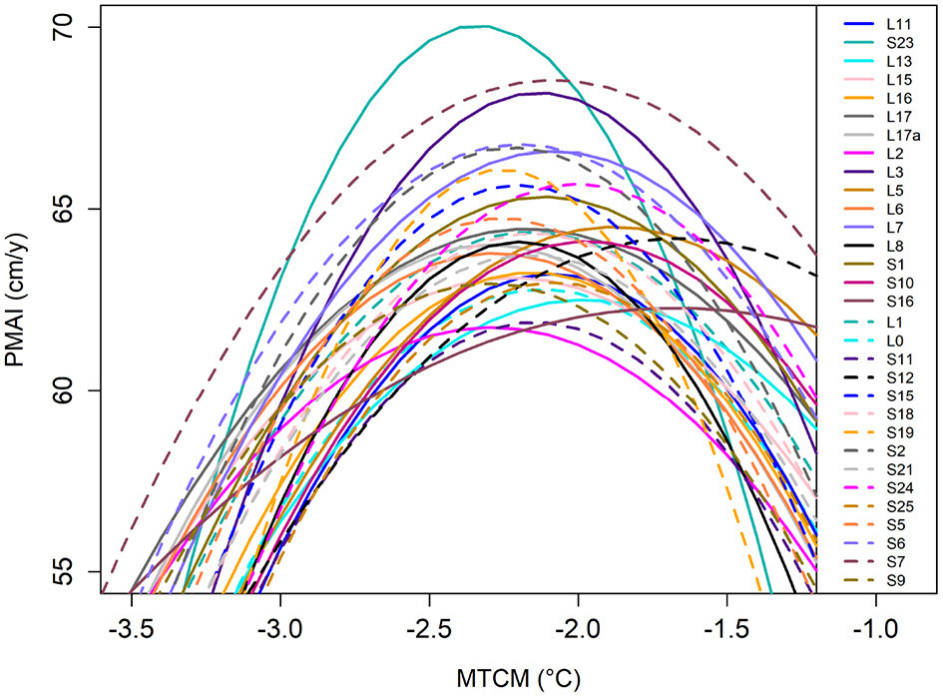
RF at the family level (solid and dashed lines). PMAIs are plotted against the MTCM of the testing sites.

In Table 1, the number of individuals per half-sib family (*N*_*b*_) varies from 35 to 120. This uneven number is explained because it is a product of natural crosses among parents; hence the families' sizes were only revealed at the pedigree reconstruction stage. In Supplementary Figure S1, we report no statistical association between *N*_*b*_ and the mean model's $R_{adj}^{2}$; however, the model's $R_{adj}^{2}$ variability is higher for families with a smaller number of offspring. At the half-sib family level, the $R_{adj}^{2}$ ranged from 0.06 to 0.64 with a median value of 0.27 (Table 1, Figure 4). All *p*-values of the RFs curve fitting were significant (*p* < 0.05), except one (genotype L2).

**Table 1:** Summary of the quadratic models for each half-sib family.

**Family**	***N*_*b*_**	$R_{adj}^{2}$	***p*-value**
L1	107	0.26	^***^
L2	47	0.06	0.091
L3	45	0.56	^***^
L5	70	0.33	^***^
L6	58	0.11	0.014
L7	46	0.23	^***^
L8	75	0.47	^***^
L10	71	0.31	^***^
L11	54	0.51	^***^
L13	53	0.21	^***^
L15	66	0.11	0.008
L16	42	0.22	0.003
L17	119	0.13	^***^
L17a	43	0.10	0.041
S1	54	0.24	^***^
S2	59	0.29	^***^
S5	45	0.32	^***^
S6	100	0.22	^***^
S7	44	0.10	0.045
S9	68	0.15	^***^
S10	35	0.33	^***^
S11	64	0.28	^***^
S12	39	0.20	0.007
S15	104	0.34	^***^
S16	70	0.09	0.016
S18	58	0.32	^***^
S19	39	0.64	^***^
S21	120	0.31	^***^
S23	37	0.42	^***^
S24	80	0.48	^***^
S25	54	0.45	^***^

*N_b_ is designating the number of individuals per family, $R_{adj}^{2}$ is the adjusted coefficient of determination*,

*** *p < 0.001*.

In Figure 4, we plotted the response curves of all families to provide an overview of the variation in PMAIs found in the data. Some families showed substantial variation in PMAIs. For example, the genotype S23 rises above all the others in PMAI, but only for a narrow range of MTCM. With lower or higher MTCM, this genotype performs poorly compared to the other families. Some families showed less steeped curves. For example, genotype S7 does not rise as high as genotype S23 but surpasses most genotypes along the whole studied MTCM gradient with a more rounded curve. Half-sib families culminate at different PMAIs, ranging from 62 to 70 cm/year, with genotypes L2/S11 and S23, respectively. Similarly, there was a difference among the MTCM optimums of the families with a range of −2.4 to −1.6°C for the genotypes S23 and S12, respectively (Figure 4).

## 4. Discussion

Phenotypic data were regressed onto random genetic and fixed site factors using the mixed linear animal genetic model. The genetic variation observed in this study resembles typical values for height and wood density in conifers (White et al., 2007), thus app. one-third of phenotypic variation is attributable to direct allelic effects. This, along with the presence of climatic gradients, is a prerequisite to efficient response function fitting, as shown in Figure 3. The choice of MTCM as our climatic gradient is supported by Foff et al. (2014), who found that cold temperature is an important limiting factor of growth in European Larch. As the GxE interactions were not significant in the present study, there is a general tendency of the genotypes to keep the same ranking across all environments (see Figure 4). However, our results showed some response functions with a clear change in rank and/or variance across the MTCM. Therefore, one may select a set of genotypes that are performing well across all sites and combine them with those, that are performing best only in specific environments. In the case of significant GxE, one may start with calculating first-order partial derivatives with respect to climate variables of planting location and provenance origin (Wang et al., 2010; Chakraborty et al., 2015).

Compared to traditional breeding trials, the proposed methodology minimizes resources for establishing the actual experiments because all activities (phenotyping, genotyping) take place in operational afforestation sites with a designated seed source. Further, uneven gametic contributions within and among the respective sites are optimally accounted for within the combined genetic evaluation protocol, i.e., multi-site animal genetic model. Unlike the traditional breeding programs relying on transfer within and among fixed-seed zones, the current approach is flexible. Seed transfer delineation is dynamic in line with particular CC development.

There are possible pitfalls of this proposed strategy that should be addressed here. Although used in many studies, the quadratic model fitting is a simplistic representation of the trait response to the environment. It assumes a physiological response that increases to a maximum value, then drops immediately (Leites et al., 2012). In reality, the curvature results from a multidimensional space of adaptive topography reflecting a specific genetic architecture of quantitative traits. The actual underlying function is likely non-linear and non-parametric. An additional limitation is related to the future adaption of the new plantations established from the offspring of the selected parents. While these would be better adapted to new climatic conditions in the short term, evaluating the long-term selection response across multiple generations is more complicated. Repeated cycles of selection would affect the environmental sensitivity depending on the functional characteristics of the reaction norms (Kolmodin et al., 2003). Optimizing the long-term methodology across multiple selection cycles should be the subject of future research.

The particular finding of our investigation can be seen as a case study demonstrating that combining *in-situ* large-scale genetic evaluation with response function methodology works. Compared to the current methodology of response functions, we are adding the opportunity to utilize the intra-population genetic variation that can further boost the adaptive response to CC. Ultimately, one would be interested in combining the provenance-based response function methodology with the presented intra-population approach. We are suggesting to optimize gene contributions from the two genetic hierarchical levels utilizing methods that were initially developed in forest tree breeding to optimize artificial selection (Lindgren et al., 1993). Assisted migration would then follow optimum contributions, thus maximizing overall adaptive response across a range of environmental conditions while maintaining sufficient levels of genetic diversity (Sáenz-Romero et al., 2021).

The suggested methodology could be practically implemented as follows. (1) identification of a common seed source representing a specific population, i.e., a provenance, (2) phenotypic evaluation followed by pedigree reconstruction (Lstibůrek et al., 2015), (3) phenotypic measurements across multiple sites combined with the pedigree in multivariate statistical analysis to predict the genetic merit of individual trees, (4) selection of principal environmental gradients influencing the studied traits, and (5) development of the individual- and population-level RFs to describe the genetic variation along prevalent environmental gradients, (6) selection of the best-adapted reforestation material accounting for genetic diversity (Funda et al., 2009), and (7) transfer of the adapted forest reproductive material to the target location.

## Data Availability Statement

The datasets presented in this study can be found in online repositories. The names of the repository/repositories and accession number(s) can be found at: https://github.com/mlstiburek/indiv_tree_response_functions.

## Author Contributions

ML and SS conceived the project. VP and DC conducted statistical analyses. VP and ML wrote the manuscript. SS, DC, JS, and HK contributed to discussions. All authors contributed to the article and approved the submitted version.

## Funding

VP was financed by the Internal Grant Agency of the Faculty of Forestry and Wood Sciences, Czech University of Life Sciences Prague. DC, HK, and SS were supported by the Austrian Research Promotion Agency (FFG) and the Cooperation Platform Forst Holz Papier (FHP) and LIECO nurseries and the Austrian Federal Forests (ÖBf). ML was supported by grant “EXTEMIT – K”, no. CZ.02.1.01/0.0/0.0/15_003/0000433 financed by OP RDE. ML and JS were supported by the Ministry of Education, Youth, and Sports program INTER-EXCELLENCE, subprogram INTER-ACTION [grant number LTAUSA19113].

## Conflict of Interest

The authors declare that the research was conducted in the absence of any commercial or financial relationships that could be construed as a potential conflict of interest.

## Publisher's Note

All claims expressed in this article are solely those of the authors and do not necessarily represent those of their affiliated organizations, or those of the publisher, the editors and the reviewers. Any product that may be evaluated in this article, or claim that may be made by its manufacturer, is not guaranteed or endorsed by the publisher.
