# Supplementary material for: Accelerating Adaptation of Forest Trees to Climate Change Using Individual Tree Response Functions
Source: Front Plant Sci. 2021 Nov 23;12:758221. doi: 10.3389/fpls.2021.758221 (PMC8650053; doi:10.3389/fpls.2021.758221)
Supplement: Supplementary file 2 [file Data_Sheet_1.docx]

**Genetic evaluation: model fit statistics**

We compared the final bivariate model B_1_ with the reduced model (B_0_; no genetic relationship matrix) using the Akaike Information Criterion (*AIC*), the Bayesian Information Criterion (*BIC*), and the restricted maximum likelihood ratio test (*LRT*), see Table S2.

We found that both *AIC* and *BIC* were smaller in B_1_, and *LRT* was highly significant (*p*-value < 0.001). These results indicate the superior predictive performance of model B_1_ compared to the reduced model B_0_.

**Table S2.** Model fit statistics of the bivariate genetic animal model.

| Model | *AIC* | *BIC* | *LRP* *p*-value |
| --- | --- | --- | --- |
| B_0_ | 8111 | 8128 |  |
| B_1_ | 7946 | 7981 | 2.2e-16 *** |

**Random forest: selection of climatic variables**

**Table S3.** Frequency of the variables’ recurrence in 10 random forest models. Each model was evaluated by the mean decrease in accuracy (%*IncMSE*) and the mean decrease in node impurity (*IncNodePurity*). For each model, the seven most important variables for each importance measure were identified and summed. The variables in bold are the most recurring ones that were used in the subsequent analysis. The symbols are *Alt* (altitude), *biol4* (temperature seasonality (standard deviation *100)), *biol9* (mean temperature of the driest quarter), *biol12* (annual precipitation), *prec5* (precipitation of May), *tmax1* (maximum temperature of January), *tmax2* (maximum temperature of February), *tmax6* (maximum temperature of June), *tmax11* (maximum temperature of November), *tmax12* (maximum temperature of December), *tmean1* (mean temperature of January), *tmean5* (mean temperature od May), *tmean9* (mean temperature of September), *tmean12* (mean temperature of December), *tmin1* (minimum temperature of January), *tmin5* (minimum temperature of May), *tmin9* (minimum temperature of September), and *tmin12* (minimum temperature of December).

| Variable name | %*IncMSE* | *IncNodePurity* | Freq |
| --- | --- | --- | --- |
| ***Alt*** | **10** | **8** | **18** |
| *biol4* | 3 | 2 | 5 |
| *biol9* | 1 | 0 | 1 |
| *biol12* | 1 | 2 | 3 |
| *prec5* | 1 | 0 | 1 |
| ***tmax1*** | **5** | **1** | **6** |
| *tmax2* | 4 | 0 | 4 |
| *tmax6* | 1 | 2 | 3 |
| *tmax11* | 1 | 0 | 1 |
| ***tmax12*** | **6** | **9** | **15** |
| ***tmean1*** | **9** | **10** | **19** |
| *tmean5* | 0 | 2 | 2 |
| *tmean9* | 2 | 3 | 5 |
| ***tmean12*** | **6** | **8** | **14** |
| ***tmin1*** | **8** | **9** | **17** |
| *tmin5* | 0 | 1 | 1 |
| *tmin9* | 2 | 3 | 5 |
| ***tmin12*** | **4** | **4** | **8** |

**Sensitivity of the response function models**

**Fig. S1.** Plot showing the linear relationship between *N_b_* and $R_{adj}^{2}$*.*
